# Low levels of free triiodothyronine are associated with risk of cognitive impairment in older euthyroid adults

**DOI:** 10.1038/s41598-023-49285-w

**Published:** 2023-12-13

**Authors:** Hao Chen, Jin Hu, Xing Yang, Quanxiang Zhou, Yuxin Hu, Xiaoyan Tang, Ji Tang, Li Zeng, Jingyuan Yang

**Affiliations:** 1 Department of Epidemiology and Health Statistics, School of Public Health, The Key Laboratory of Environmental Pollution Monitoring and Disease Control, Guizhou Medical University, Guiyang, China; 2 School of Medicine and Health Management, Guizhou Medical University, Guiyang, China; 3 The Third People’s Hospital of Guizhou Province, Guiyang, China; 4 Department of Clinical Medicine, Qinnan Medical College for Nationalities, Qiannan, China

**Keywords:** Endocrinology, Health care, Risk factors

## Abstract

Accumulated evidence showed that thyroid diseases induced cognitive decline. However, the relationship between thyroid hormones (THs) and cognition in older euthyroid people is still unclear. Our study aimed to estimate the association between THs within the euthyroid range and cognition in community-dwelling older adults in China. Data were extracted from a cohort study on the health status of rural older adults from the Guizhou province in China (HSRO). Serum thyroid-stimulating hormone (TSH), free thyroxine (FT_4_), and free triiodothyronine (FT_3_) were measured using the electrochemiluminescence immunoassay. Cognitive function was evaluated by the Mini-Mental State Examination (MMSE). Linear regression and a binary logistic regression model were used to explore the relationship between THs and cognition in euthyroidism (TSH level of 0.27 ~ 4.20mIU/L). A total of 957 euthyroidism individuals were included in this study, with a mean (SD) age of 71.34 (6.35) years. In individuals with euthyroidism, serum TSH and FT_3_ levels were positively associated with cognition (*TSH:β* = *0.06, 95% CI*  *0.01* ~ *0.11, P* = *0.03; FT*_*3*_*:β* = *0.07, 95% CI*  *0.01* ~ *0.12, P* = *0.01*); and serum FT_3_ and TSH levels were significantly associated with cognitive domains (*P* < *0.05*). Further, euthyroid individuals in the lowest serum FT_3_(*OR* = *1.96; 95% CI 1.27* ~ *3.03*) quartile had a twofold increased risk of cognitive impairment compared to those in the highest quartile after adjusting for potential confounding factors. These findings suggested that low levels of FT_3_ could be an independent risk factor for cognitive impairment in older euthyroid adults. Additionally, a positive linear association exists between serum FT_3_ levels and cognitive domains (such as immediate memory, language, and attention). Further studies are needed to determine the underlying mechanisms and the community significance of these findings.

## Introduction

Due to the aging population, the incidence of Alzheimer’s Disease (AD) will increase to over 100 million people worldwide by 2050, which implies that 1 in every 85 individuals in the world may suffer from the disease^1^. No treatment is available to slow or stop the deterioration of brain cells in AD. The increasing prevalence of AD will lead to a huge burden for society and families^1^. Early detection of risk factors and incidence is a critical strategy in the prevention of AD. Thyroid hormones (THs) are essential for the development of the central nervous system (CNS) and for regulating metabolism, neurogenesis, myelination, and cellular repair in the brain throughout the lifespan of an individual^2–4^. Both in vitro and in vivo thyroid function studies have indicated that abnormal THs levels could lead to impaired cognitive function^5,6^. Studies derived from clinical institutions and patients also showed an association between thyroid function and cognitive function^7–10^.

Nevertheless, some observational studies on individuals in the community have yielded controversial results in older adults. A prospective investigation showed no significant association between subclinical thyroid d ysfunction and cognitive decline^11^. Similarly, a collaborative project in 25 cohorts did not support the association between thyroid dysfunction and impairment in cognitive function^12^. A cohort study found that thyroid disorders were associated with AD development^13^. Observational studies have yielded inconsistent results, which might be attributed to the unstable manifestations of that thyroid disorders among older adults. Some of these studies were limited by heterogeneity in definitions of thyroid dysfunction, while in others, subclinical thyroid dysfunction in older adults might return to normal over time^14^. Meanwhile, researchers have also studied the impact of changes in the levels of THs within the normal reference range on cognitive function, particularly in older adults. Such data could provide new insights into the association of levels of THs with cognitive decline^15^.

Some studies have found an association between cognition and thyroid indicators (thyroid-stimulating hormone (TSH) or free thyroxine (FT_4_)) within the normal reference range^16–18^. However, a cohort study by Samuels et al.^19^ discovered that minor thyroid hormone variations did not affect the cognition of community-dwelling older adults. Notably, these investigations primarily focused on examining the influence of a single thyroid indicator on cognitive function. Owing to the way the THs affect each other via the hypothalamus-pituitary-thyroid (HPT) axis, there is a lack of comprehensive exploration of the relationship between THs and cognitive function in community-dwelling older adults. Hence, it is necessary to observe the association between thyroid hormones in the euthyroid range and cognitive function in older populations. In this study, we used data from cross-sectional studies to investigate the aging population in a community in Guizhou province.

## Results

A total of 1235 subjects were included in this study for thyroid function assessment. Of these, 957 (77.49%) individuals had euthyroidism. The demographic characteristics and MMSE scores of the euthyroid participants are shown in Table 1. The mean (SD) age of the euthyroid participants was 71.34 (6.35) years. Women had lower cognitive scores than men (*P* < 0.001). Further, we observed that MMSE scores significantly declined with increasing age (*P* < 0.001). Participants with hypertension, depression, or anxiety, and those who were single or widowed/divorced had significantly lower MMSE scores (*P* < 0.001). Moreover, individuals from currently smoking and currently drinking groups got significantly higher MMSE scores (*P* < 0.001). Likewise, serum levels of TSH and THs differed by age, gender, and education level, as shown in Table S1. The fundamental demographic details of PHQ-2 and GAD-2 in Table S2.

**Table 1:** Characteristics of the distribution of MMSE scores in older adults with euthyroidism.

Characteristics	Participants (%)	MMSE
Gender		
Male	429 (44.82)	23.54 ± 4.62
Female	528 (55.18)	18.85 ± 5.30
* T(P)*		**14.43 (< 0.001)**
Age (year)		
60 ~	415 (43.36)	22.36 ± 4.99
70 ~	436 (45.56)	20.50 ± 5.42
80 ~	106 (11.08)	17.29 ± 5.96
* F(P)*		**41.50 (< 0.001)**
Education (level)		
Illiteracy	728 (76.07)	19.61 ± 5.36
Primary	142 (14.84)	24.89 ± 3.32
Junior/high	87 (9.09)	25.76 ± 3.66
* F (P)*		**111.69 (< 0.001)**
Marital status		
Single	3 (0.31)	19.67 ± 8.51
Married	601 (62.80)	21.74 ± 5.19
Widowed/divorced	353 (36.89)	19.62 ± 5.81
* F(P)*		**17.02 (< 0.001)**
Hypertension		
Yes	567 (59.25)	20.33 ± 5.57
No	390 (40.75)	21.85 ± 5.33
* t(P)*		** − 4.23 (< 0.001)**
Smoking		
Yes	272 (28.42)	23.14 ± 4.68
No	685 (71.58)	20.04 ± 5.57
* t(P)*		**8.37 (< 0.001)**
Drinking		
Yes	259 (27.06)	21.61 ± 5.71
No	698 (72.94)	20.71 ± 5.43
* t(P)*		**2.26 (0.02)**
Anxiety symptoms		
Yes	136 (14.21)	19.72 ± 5.30
No	821 (85.79)	21.12 ± 5.51
* t(P)*		** − 2.65 (0.008)**
Depression symptoms		
Yes	135 (14.11)	19.99 ± 5.32
No	822 (85.89)	21.11 ± 5.54
* t(P)*		** − 2.20 (0.03)**

Bold type indicates that t-test or ANOVA was used to test the MMSE scores between demographic data that the correlation is significant (P < 0.05). Data are presented as mean values and standard deviations for continuous variables or percentages (%) for categorical variables.

*MMSE* mini–mental state examination.

**Figure 1:**
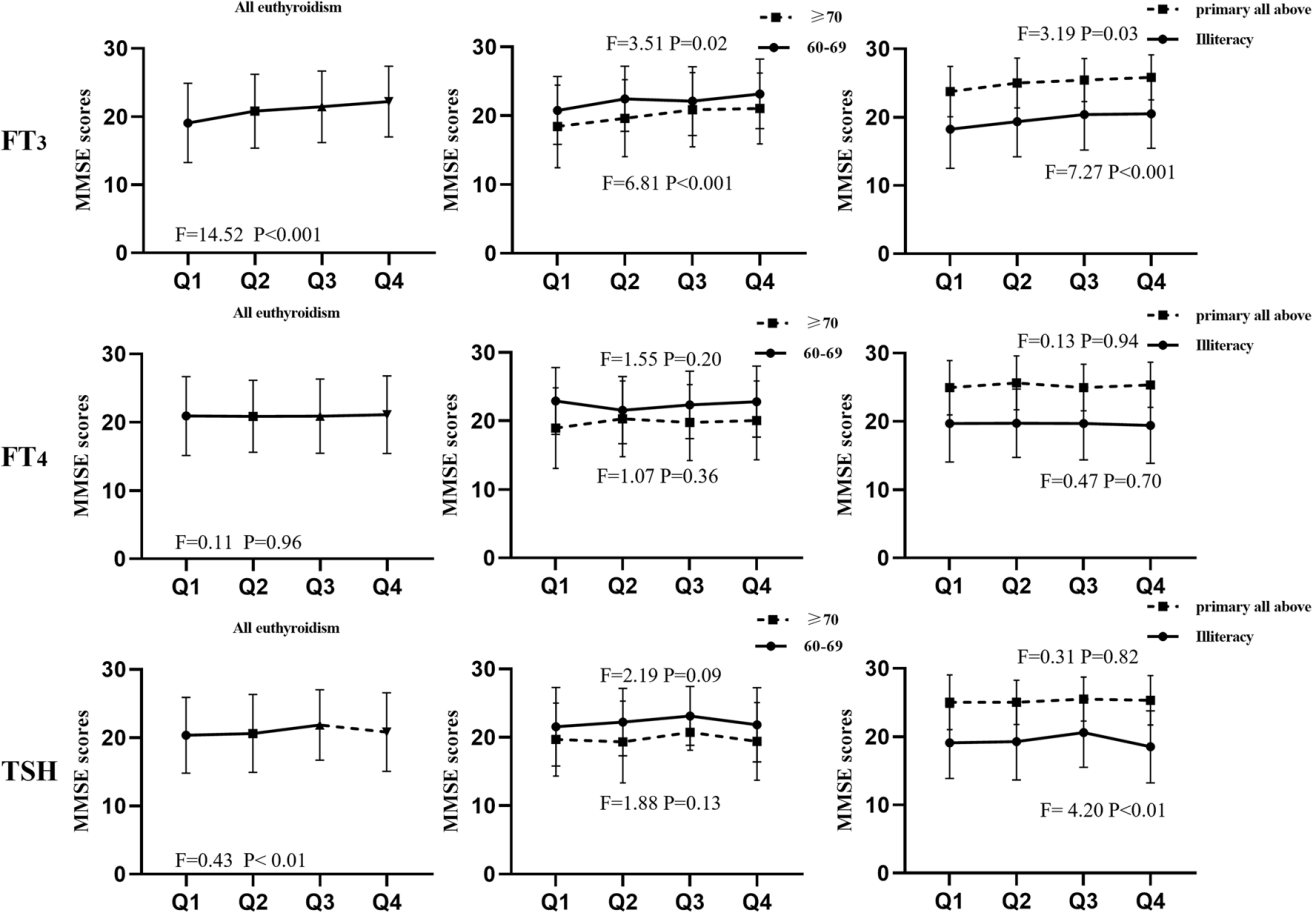
The MMSE scores of different serum thyroid hormone levels concentrate in euthyroidism. *TSH* thyroid-stimulating hormone, *FT*_4_ free thyroxine, *FT*_3_ free triiodothyronine, *Q* quartile.

As shown in Fig. 1, we observed statistically significant differences in MMSE scores with varying FT_3_ concentrations. Individuals with lower FT_3_ concentrations had lower MMSE scores. The quartiles for the TSH concentration group showed statistically significant differences in MMSE scores for concentrations in Q3 and below. Further, we assessed the correlations between various thyroid markers and cognitive performance in different thyroid statuses, revealing that serum FT_3_ concentration was independently associated with cognitive function in euthyroidism (Table 2).

**Table 2:** Correlation between MMSE scores and thyroid indicators.

Groups	N (%)	Correlation coefficients (95% CI)		
		TSH	FT_4_	FT_3_
All participants	1235	0.08 (0.02–0.13)^a^	0.04 (− 0.02–0.10)	0.19 (0.14–0.25)^a^
Thyroid status				
Euthyroidism	957 (77.49)	0.11 (0.05–0.18)^a^	0.03 (− 0.03–0.10)	0.20 (0.14–0.26)^a^
SCH	188 (15.22)	0.01 (− 0.14–0.15)	0.001 (− 0.14–0.15)	0.14 (− 0.01–0.28)
SCHper	66 (5.34)	0.01 (− 0.37–0.40)	0.19 (− 0.27–0.61)	0.23 (− 0.19–0.59)
Hypothyroidism	24 (1.94)	0.15 (− 0.11–0.40)	− 0.09 (− 0.32–0.18)	0.18 (− 0.07–0.43)

*TSH* thyroid-stimulating hormone, *FT4* free thyroxine, *FT3* free triiodothyronine, *SCH* subclinical hypothyroidism, *SCHyper* subclinical hyperthyroidism, *CI* confidence interval.

^a^Indicates the correlation is significantly (*P* < 0.05).

**Figure 2:**
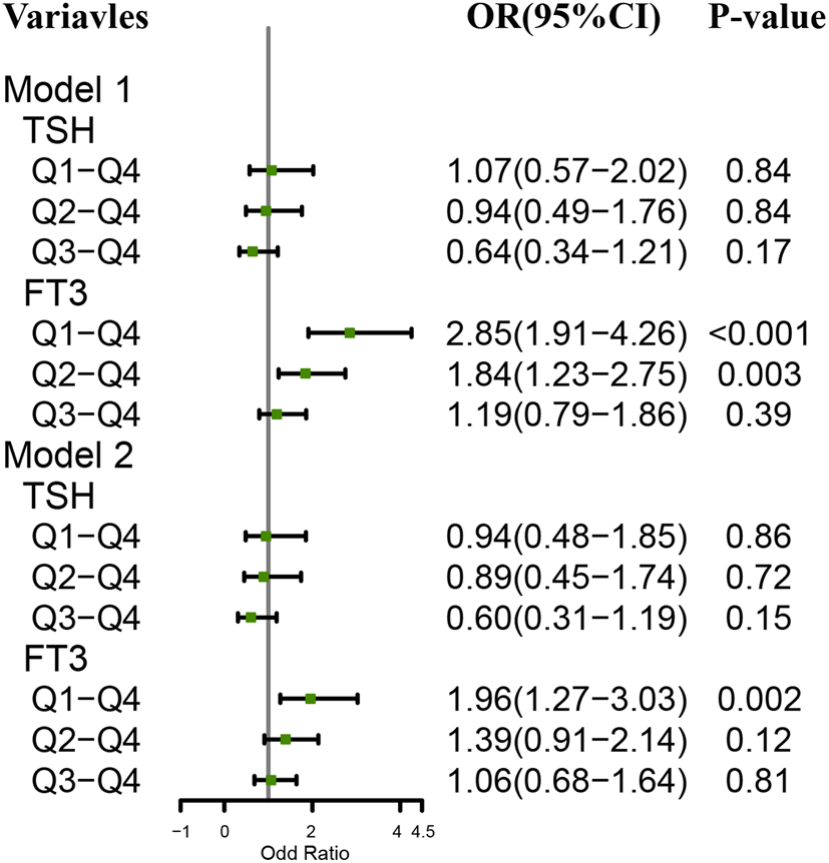
Cognitive impairment and quartiles of thyroid indicators. *TSH* thyroid-stimulating hormone, *FT*_3_ free triiodothyronine, *Q* quartile, *CI* confidence interval. Q4 as reference. Model 1 is not adjusted; Model 2 is adjusted for age, gender, education, marital status, hypertension, smoking, drinking, anxiety, and depression.

As shown in Table 3, the serum levels of FT_3_ (*β* = 0.07; 95% CI 0.01 ~ 0.12) and TSH (*β* = 0.06; 95% CI 0.01 ~ 0.11) were associated with the MMSE score. Further subgroup analysis revealed that in the TSH levels (Q4) group, FT_3_ was associated with MMSE scores, while in the high FT_3_ levels (Q4) group, TSH was not statistically associated with MMSE scores. Moreover, as shown in Fig. 2, the lowest level of FT_3_(*OR* = 1.96; 95% CI 1.27 ~ 3.03) was an independent risk factor for cognitive impairment after adjusting for age, gender, anxiety, depression, education level, and other confounding variables. The distribution of characteristics of all subjects assessed for thyroid function and the proportion of cognitive impairment in different groups are shown in Supplementary Tables S3, S4, and S5. Further, we found that serum FT_3_ levels were associated with the cognitive domains of immediate memory and language, while serum TSH levels were found to have a weak positive association with immediate memory and attention (Table S6).

**Table 3:** Associations between thyroid indexes and MMSE scores in euthyroidism.

Variables	β (95% CI)	*P* value
Model 1		
FT_3_	0.15 (0.09–0.21)	< 0.001
FT_4_	0.02 (− 0.15–0.18)	0.64
TSH	0.11 (0.05–0.17)	< 0.001
Model 2		
FT_3_	0.07 (0.01–0.12)	0.01
FT_4_	0.01 (− 0.05–0.06)	0.81
TSH	0.06 (0.01–0.11)	0.03
TSH and FT_3_ subgroup analysis		
TSH levels = Q4		
FT_3_	0.32 (0.04–0.59)	0.02
FT_4_	0.07 (− 0.24–0.39)	0.65
TSH	0.04 (− 0.27–0.35)	0.80
FT_3_ levels = Q4		
FT_3_	− 0.09 (− 0.22–0.03)	0.13
FT_4_	0.05 (− 0.08–0.17)	0.43
TSH	0.05 (− 0.08–0.17)	0.46

Model 1 represents the unadjusted model. Model 2 is adjusted for age, gender, education, marital status, hypertension, smoking, drinking, anxiety, and depression. Q4 is the highest level of the quartile.

*TSH* thyroid-stimulating hormone, *FT*_4_ free thyroxine, *FT*_3_ free triiodothyronine, *CI* confidence interval.

## Discussion

In this observational study of Chinese community-dwelling older euthyroid adults, we found a positive and a weak positive correlation of serum FT_3_ and TSH levels with cognition, respectively. Further, low FT_3_ levels were found to double the risk for cognitive impairment. Our results suggested that minor changes in thyroid indicators could impact cognition function even in older euthyroid adults. To the best of our knowledge, this was the first study that focused on the relationship between THs and cognition in older euthyroid adults in a Chinese community.

Previous studies have primarily focused on exploring the relationship between thyroid dysfunction and cognition. However, thyroid dysfunction has been found to be less prevalent in older communities, which might be attributed to the heterogeneity in definitions of thyroid dysfunction^20^. This might have led to subclinical hypothyroidism and hyperthyroidism not being found to be associated with cognitive function^12^. While investigating the association between cognition and thyroid function, we used continuous measurements to address the above-mentioned limitations and received the same results. Our study, in line with a longitudinal investigation in Brazil^21^, found no significant correlation between FT_4_ and cognitive function. The nuanced actions of thyroid hormones are likely mediated through interactions with specific thyroid nuclear receptors, as FT_4_ and FT_3_ are secreted by the thyroid gland and transported to target cells via distinct serum proteins. Given that FT_3_ is the active form of the hormone, the existing literature supports the notion that FT_3_, rather than FT_4_, is more likely to instigate changes in cognitive function^22^. In contrast, a clinical study involving individuals with normal thyroid function reported an association between serum FT_4_ levels and Alzheimer’s disease pathology^23^, contradicting our findings. This discrepancy may arise from the limited subject pool in the clinical study, leading to a narrow distribution of FT_4_ levels. Additionally, an alternative mechanism underlying the association between thyroid hormones and cognitive decline could be the dynamic changes in the expression of these hormones throughout an individual’s lifespan in response to the evolving needs of various organs and the aging process^24,25^. These factors deserve further exploration in future studies. Additionally, our findings revealed that when TSH levels are high (Q4), there is an observed association between FT_3_ and MMSE scores. Conversely, when FT_3_ levels are high (Q4), TSH is not found to be statistically associated with MMSE scores. The relationship between FT_3_ and TSH in older adults with euthyroidism involves a dynamic interplay. Low FT_3_ concentrations may lead to increased TSH secretion, and changes in TSH levels can also influence FT_3_ concentrations. The findings suggested that the influence of thyroid hormones on cognitive function may vary based on hormone concentrations. Future research should explore the temporal dynamics and bidirectional influences within the hypothalamus–pituitary–thyroid axis and their specific impact on cognitive function.

In our study, we observed no significant association between thyroid-stimulating hormone levels and cognitive impairment, and the lowest concentration of FT_3_ was linked to a twofold risk of cognitive impairment. These findings were in line with a single-center study that evaluated patients with dementia^10^, which may be attributed to the altered activity of deiodinase type 2 (D2), expressed in various tissues, which regulates the conversion of T_4_ to T_3_ in the central nervous system. D2 activity could be considered as another possible mechanism contributing to the association between thyroid hormones and cognitive decline. Further investigations are warranted to delve into this mechanism and establish a more comprehensive understanding. Similarly, our results align with cohort studies that found no correlation between normal range or mild changes in TSH and cognitive dysfunction^26^. This concordance may be attributed to our study population, consisting of elderly individuals with normal thyroid function. In post-mortem and mono-center studies, AD patients have been found to exhibit low serum levels of FT_3_, which was consistent with our findings^10,25^. Similarly, in older euthyroid adults, a previous cross-sectional study found that the risk factors for cognitive impairment were positively associated with FT_3_ levels^27^. Our study contributes to the growing evidence associating FT_3_ levels with cognition in older euthyroid adults, suggesting a nuanced interplay between TSH and FT_3_ within the normal thyroid function range. Furthermore, we also found that TSH and FT_3_ levels were associated with the cognitive domain of immediate memory. Previous observable research found an association between TSH levels in the normal range and memory performance^28,29^. The prefrontal cortex is a brain region of great importance for memory^30^. A study on older mice with significantly impaired learning and memory showed lower serum levels of FT_3_ and higher levels of SNAP-25 and MUNC18-1 in the frontal lobe of the mice^31^. This finding held potential significance in enhancing our understanding of the mechanisms linking TSH and FT_3_ levels with cognitive domains in euthyroidism.

The current study had several limitations. Firstly, this is a cross-sectional epidemiological survey in rural Guizhou province, with no evidence of age-related cognitive changes in the selected cohort. Secondly, this study lacks the exploration of inflammatory markers. All the associations might have arisen due to the modulation of inflammatory factors produced during the various non-thyroidal diseases. Thirdly, the lack of data regarding serum total T_3_, total T_4_, thyroid-specific antibodies, and enzymes involved in the conversion of T_4_ to T_3_ might have limited the interpretation of our findings. There is a need for a more comprehensive assessment to elaborate on the mechanisms underlying the impact of thyroid hormones on the central nervous system. Fourthly, the MMSE is a brief assessment of overall cognitive function and may not be sensitive to the more subtle changes across our lifespan.

In conclusion, euthyroidism is mostly neglected by researchers. However, this state is also essential in the community, particularly among older adults with cognitive decline. Our findings from older adults in the Chinese community suggested an independent relationship between serum FT_3_ levels and cognitive function within normal thyroid function, with low levels of FT_3_ associated with the risk of cognitive impairment. Additionally, TSH and FT_3_ levels also impacted the cognitive domain of immediate memory. As these findings are derived from cross-sectional data, further confirmation is needed from a longitudinal exploration of impact of FT_3_ supplementation on the cognitive function of euthyroid older adults.

## Materials and methods

### Study design and participants

This is a cross-sectional analysis of the baseline survey data obtained from the cohort study on the health status of rural older adults from the Guizhou province in China (HSRO). The data were obtained using multistage cluster sampling. A total of 12 villages in the province were selected, and the baseline survey was conducted from July to August 2019. The inclusion criteria included community-dwelling volunteers aged ≥ 60 years who were long-term residents of the area (at least > 6 months). A total of 1795 older adults were enrolled in this study. Among them, 560 individuals were excluded for the following reasons: (i) did not cooperate in the blood sample collection, (ii) did not have normal communication skills, (iii) had been diagnosed with severe mental illness by the medical center (including severe depression and anxiety), and (iv) had taken drugs that affected thyroid function, such as levothyroxine and thyroxine. Thus, 1235 community-dwelling older adults were included in the thyroid function assessment analysis, of whom 957 were euthyroid individuals. The study was approved by the Ethics Committee of Guizhou Medical University, and all the participants signed informed consent.

### Analyses

#### Assessment of thyroid function

Blood samples were collected the morning after an overnight fast. Serum levels of TSH, free thyroxine (FT_4_), and free triiodothyronine (FT_3_) were determined using the Roche Cobase601 automated chemiluminescent immunoassays (Roche Group, Switzerland). The reference ranges provided by the manufacturer were as follows: FT_3_, 3.10 ~ 6.80 pmol/L; FT_4_, 12.00 ~ 22.00 pmol/L; and TSH, 0.27 ~ 4.20 mIU/L.

Participants were categorized based on TSH, FT_3_, and FT_4_ concentrations, the normal thyroid function also called euthyroidism (TSH = 0.27 ~ 4.20mUI/L); overt hypothyroidism (TSH > 4.20 mIU/L and FT_4_ < 12.00 pmol/L); subclinical hypothyroidism (TSH > 4.20mIU/L and FT_4_ = 12.00 ~ 22.00 pmol/L); subclinical hyperthyroidism (TSH < 0.27 mIU/L, FT_4_ = 12.00 ~ 22.00 pmol/L, and FT_3_ = 3.10 ~ 6.80 pmol/L) and overt hyperthyroidism (TSH < 0.27 mIU/L, FT_4_ > 22.00 pmol/L, and FT_3_ > 6.80 pmol/L)^32^.

#### Assessment of cognitive function

The mini-mental state examination (MMSE) score was used to assess the cognitive function of participants^33^. MMSE results can quickly reflect a global cognition in clinical, research, and community settings. The test comprises 11 items divided into five domains: orientation, immediate memory, attention, language, and delayed recall. Trained investigators recorded the participants’ responses while observing their behavior and then summed the scores assigned to each question. The 30-point questionnaire contains a total of 30 test questions. For each correct response, the participants would score 1 point; thus, each participant could obtain a total score of 0 to 30 points. Cognitive impairment was defined as MMSE score of ≤ 17 for illiterate participants,  ≤ 20 for those with primary school education and below, and ≤ 24 for those with junior high school education and above^34^.

#### Covariates

In this study, we examined several demographic characteristics of the participants, including gender, marital status (single, married, and widowed/divorced), and education (illiteracy, primary, and high level), age (age groups of 60–69, 70–79, ≥ 80 years). Based on their smoking status, the participants were divided into two categories: currently smoking (defined as a total of > 100 cigarettes smoked in the past year) or not smoking (including quitting smoking (defined as quitting for > 6 months) and never smoking). The drinking category was divided into 2 categories: currently drinking (defined as drinking on an average of ≥ 1 days per week in the past year) or not drinking (including never/occasional drinking for > 6 months). Hypertension was assessed through self-report; participants needed to answer the question, “Have you ever been told by a doctor, nurse, or another health professional that you have hypertension?” Responses were grouped into two categories: Yes and no.

### Depressive symptoms assessment

Utilizing the Patient Health Questionnaire-2 (PHQ-2), the evaluation of depressive symptoms was conducted. Comprising two items, this questionnaire probes into the frequency of depressive mood and feelings of loss over the preceding two weeks. Response options, including "not at all", "a few days", "more than half of the days", and "almost every day", are associated with scores of 0, 1, 2, and 3, respectively. Consequently, PHQ-2 scores span from 0 to 6, with a score of 3 or more indicating the potential presence of a depressive disorder^35^.

### Anxiety symptoms assessment

For the assessment of anxiety symptoms, the Generalized Anxiety Disorder 2-Item Scale (GAD-2) is employed as a concise and user-friendly tool designed for the initial screening of generalized anxiety disorder. This scale consists of two items that investigate the frequency of anxiety over the past two weeks. Response options, ranging from "not at all" to "almost every day", are correspondingly assigned scores of 0, 1, 2, and 3. Consequently, GAD-2 scores range from 0 to 6, and a score of 3 or higher suggests the potential presence of an underlying anxiety disorder^36^.

### Statistical analysis

All statistical analyses were performed using SPSS for Windows version 22.0 (IBM Corp., Armonk, NY, USA). MMSE scores and, THs were analyzed by quartile grouping in order to observe cognitive differences. Spearman’s correlation coefficients were calculated to estimate the association of MMSE scores with thyroid hormones. Bootstrapping was used to obtain 95% confidence intervals (CIs). Multiple linear regression and binary logistic regression analysis were used to assess the relationship between THs and cognitive function (domains) after adjustment for age, gender, marriage, and other relevant variables (smoking, drinking, anxiety, and depression). The difference between all subjects and euthyroidism was assessed and shown in the appendix. All statistical tests were 2-sided and the difference were considered significant at *P* < 0.05.

### Ethics approval and consent to participate

Written informed consent was obtained from each participant before any study procedure was initiated, and the collection of data on human subjects was approved by the medical ethics committee of Guizhou Medical University (approval No. 2018-092). All methods in this study were performed in accordance with the guidelines of the Declaration of Helsinki.

### Supplementary Information

Supplementary Tables.

## Data Availability

The datasets that support the findings of this study are available on request from the corresponding author (Jingyuan Yang, e-mail: yang8880@sina.com. The data is not publicly available due to privacy or ethical restrictions.

## Abbreviations

AD: Alzheimer’s disease
THs: Thyroid hormones
TSH: Thyroid-stimulating hormone
FT4: Free thyroxine
FT3: Free triiodothyronine
MMSE: The mini-mental state examination (MMSE)
