# Supplementary material for: Low levels of free triiodothyronine are associated with risk of cognitive impairment in older euthyroid adults
Source: Sci Rep. 2023 Dec 13;13:22133. doi: 10.1038/s41598-023-49285-w (PMC10719249; doi:10.1038/s41598-023-49285-w)
Supplement: Supplementary file 1 — Supplementary Tables. [file 41598_2023_49285_MOESM1_ESM.pdf]

Low levels of free triiodothyronine are associated with risk of cognitive impairment in older euthyroid adults

Hao Chen[1,3], Jin Hu[1], Xing Yang[2], Quanxiang Zhou[4], Yuxin Hu[1], Xiaoyan Tang[1], Ji Tang[1], Li Zeng[1], Jingyuan Yang[1*]

*Correspondence: yangjingyuan@gmc.edu.cn

1. Department of Epidemiology and Health Statistics, School of Public Health, The Key Laboratory of Environmental Pollution Monitoring and Disease Control, Guizhou Medical University, Guiyang, China.

2. School of medicine and health management, Guizhou Medical University, Guiyang, China.

3. The Third People's Hospital of Guizhou Province, Guiyang, China.

4. Department of Clinical Medicine, Qinnan Medical College for Nationalities, Qiannan, China.

(Supplementary files)

Table S1 The distribution of thyroid function tests among the demographic

characteristics in euthyroidism.

Characteristics	Participants (n)	TSH [a]	FT_4 (pmol/L)	FT_3 (pmol/L)
Euthyroidism	957	0.42±0.15	15.84±2.10	4.41±0.70
Gender				
Male	429	0.42±0.14	16.07±2.31	4.50±0.63
Female	528	0.41±0.15	15.66±1.91	4.33±0.75
t(P)		1.03(0.30)	**2.98(<0.01)**	**3.79(<0.001)**
Age（Year）				
60~	415	0.43±0.15	15.65±2.08	4.49±0.58
70~	436	0.40±0.14	16.00±2.08	4.36±0.65
80~	106	0.41±0.15	15.93±2.25	4.21±1.15
F(P)		**7.39(<0.01)**	3.03(0.05)	**8.36(<0.001)**
Education（level）				
Illiteracy	728	0.41±0.15	15.76±2.06	4.37±0.73
Primary	142	0.43±0.15	16.36±2.22	4.48±0.54
Junior/High	87	0.45±0.15	15.61±2.13	4.55±0.69
F(P)		2.92(0.06)	**5.32(0.01)**	**3.27(0.04)**
Marital status				
Single	3	0.40±0.05	16.06±3.12	4.69±0.46
married	601	0.42±0.15	15.85±2.01	4.44±0.72
Widowed/Divorced	353	0.41±0.15	15.82±2.13	4.35±0.67
F(P)		0.16(0.85)	0.05(0.94)	2.02(0.13)
Hypertension				
Yes	567	0.42±0.15	15.95±2.16	4.41±0.72
No	390	0.42±0.15	15.68±2.01	4.41±0.68
t(P)		-0.15(0.89)	1.93(0.05)	-0.04(0.96)
Smoking				
Yes	272	0.42±0.14	15.98±2.24	4.48±0.59
No	685	0.42±0.15	15.78±2.05	4.38±0.74
t(P)		0.67(0.50)	1.26(0.21)	1.94(0.05)
Drinking				
Yes	324	0.42±0.15	15.98±2.24	4.46±0.78
No	911	0.42±0.15	15.79±2.05	4.39±0.67
t(P)		0.40(0.69)	1.22(0.22)	1.51(0.13)
Anxiety Symptoms				
Yes	136	0.40±0.16	15.46±1.89	4.34±0.58
No	821	0.42±0.15	15.90±2.13	4.42±0.72
t(P)		-1.87(0.06)	**-2.30(0.02)**	-1.22(0.22)
Depression Symptoms				
Yes	135	0.41±0.15	15.55±2.06	4.38±0.57
No	822	0.42±0.15	15.89±2.11	4.41±0.72
t(P)		-1.06(0.29)	-1.76(0.08)	-0.51(0.61)

TSH, Thyroid-stimulating hormone; FT_4, Free thyroxine; FT_3, Free triiodothyronine.

[a] Indicates the log-transformed of serum TSH.

Bold type indicates that t-test or ANOVA was used to test the MMSE scores between demographic data that the correlation is significant ($P < 0.05$).

Data are presented as mean values and standard deviations for continuous variables.

Table S2 Distribution of PHQ-2 and GAD-2 scores in the Study Population

	Participants （%）	Scores
Anxiety Symptoms (GAD-2)		
Total		0.99±1.67
Yes	136(14.21)	4.57±1.25
No	821(85.79)	0.40±0.73
Depression Symptoms (PHQ-2)		
Total		1.00±1.63
Yes	135(14.11)	4.44±1.24
No	822(85.89)	0.44±0.76

Table S3 The distribution of MMSE scores in the characteristics of all participants.

Characteristics	Participants（%）	MMSE
Gender		
Male	523(42.35)	23.38±4.72
Female	712(57.65)	19.11±5.33
T(P)		**14.87(＜0.001)**
Age（Year）		
60~	551(44.62)	22.31±4.97
70~	545(44.13)	20.49±5.34
80~	139(11.25)	17.07±6.00
F(P)		**58.32(＜0.001)**
Education（level）		
Illiteracy	939(76.03)	19.58±5.33
Primary	179(14.49)	24.83±3.36
Junior/High	117(9.47)	25.69±3.69
F(P)		**144.69(＜0.001)**
Marital status		
Single	7(0.57)	18.57±5.26
married	773(62.60)	21.80±5.15
Widowed/Divorced	445(36.03)	19.45±5.76
F(P)		**27.91(＜0.001)**
Hypertension		
Yes	736(59.59)	20.41±5.54
No	499(40.40)	21.67±5.36
t(P)		**-3.96(＜0.001)**
Smoking		
Yes	335(27.13)	23.18±4.72
No	900(72.87)	20.08±5.53
t(P)		**9.77(＜0.001)**
Drinking		
Yes	324(26.23)	21.74±5.56
No	911(73.77)	20.63±5.45
t(P)		**3.32(0.002)**
Anxiety		
Yes	178(14.41)	19.72±5.30
No	1057(85.59)	21.12±5.51
t(P)		**-3.14(0.002)**
Depression		
Yes	173(14.01)	19.75±5.22
No	1062(85.99)	21.11±5.52
t(P)		**-3.04(0.002)**
Thyroid status		
Euthyroidism	957(77.49)	21.00±5.52
SCH	188(15.22)	21.20±5.48
SCHper	66(5.34)	20.66±5.25

Overt hypothyroidism	24(1.94)	19.9±5.30
F（*P*）		1.07(0.36)

MMSE, Mini–Mental State Examination. SCH, Subclinical hypothyroidism; SCHyper, Subclinical hyperthyroidism.

Bold type indicates that t-test or ANOVA was used to test the MMSE scores between demographic data that the correlation is significant (P < 0.05).

Data are presented as mean values and standard deviations for continuous variables or percentages (%) for categorical variables.

Table S4 The distribution of thyroid function tests among the demographic

characteristics of all participants

Characteristics	Participants (n)	TSH [a]	FT_4 (pmol/L)	FT_3 (pmol/L)
All participants	1235	0.52±0.29	15.34±2.40	4.36±0.69
Gender				
Male	523	0.51±0.28	15.65±2.54	4.46±0.62
Female	712	0.53±0.29	15.12±2.27	4.28±0.72
t(P)		-1.07(0.29)	3.80(<0.001)	**4.26(<0.001)**
Age（Year）				
60~	551	0.55±0.27	15.20±2.24	4.46±0.57
70~	545	0.50±0.28	15.50±2.41	4.32±0.65
80~	139	0.54±0.36	15.40±2.92	4.11±1.06
F(P)		**4.9(0.01)**	2.12(0.12)	**15.94(<0.001)**
Education（level）				
Illiteracy	939	0.52±0.28	15.30±2,38	4.33±0.71
Primary	179	0.54±0.29	15.79±2.41	4.43±0.54
Junior/High	117	0.58±0.31	15.08±2.52	4.48±0.68
F(P)		2.99(0.05)	**3.99(0.02)**	**3.66(0.03)**
Marital status				
Single	7	0.73±0.34	15.50±2.87	4.57±0.47
married	773	0.52±0.28	15.40±2.30	4.40±0.69
Widowed/Divorced	445	0.52±0.30	15.26±2.56	4.28±0.68
F(P)		1.97(0.14)	0.46(0.64)	**5.21(<0.01)**
Hypertension				
Yes	737	0.53±0.29	15.44±2.50	4.36±0.71
No	499	0.52±0.28	15.22±2.25	4.36±0.65
t(P)		0.18(0.86)	1.57(0.12)	-0.24(0.81)
Smoking				
Yes	335	0.52±0.27	15.60±2.41	4.44±0.59
No	900	0.53±0.29	15.25±2.39	4.33±0.72
t(P)		-0.64(0.52)	**2.27(0.02)**	**2.54(0.01)**
Drinking				
Yes	324	0.51±0.28	15.56±2.52	4.41±0.76
No	911	0.53±0.29	15.27±2.36	4.34±0.66
t(P)		-0.98(0.33)	1.84(0.06)	1.50(0.13)
Anxiety				
Yes	178	0.52±0.29	15.00±2.16	4.29±0.56
No	1057	0.53±0.29	15.41±2.44	4.37±0.71
t(P)		-0.42(0.68)	**-2.10(0.04)**	-1.37(0.17)
Depression				
Yes	173	0.52±0.29	15.09±2.37	4.33±0.58
No	1062	0.52±0.29	15.39±2.41	4.36±0.70
t(P)		-0.26(0.80)	-1.55(0.12)	-0.7(0.48)

TSH, Thyroid-stimulating hormone; FT_4, Free thyroxine; FT_3, Free triiodothyronine.

[a] Indicates the log-transformed of serum TSH.

Bold type indicates that t-test or ANOVA was used to test the MMSE scores between demographic data that the correlation is significant (P < 0.05).

Data are presented as mean values and standard deviations for continuous variables.

Table S5 Associations between thyroid indicators and MMSE scores in thyroid status

Variables	All	Euthyroidism	Subclinical Hypothyroidism	Subclinical Hyperthyroidism	Hypothyroidism
	β (*95%CI*)	β (*95%CI*)	β (*95%CI*)	β (*95%CI*)	β (*95%CI*)
FT_3	0.07(0.02~0.12) [a]	0.06(0.01~0.12) [a]	0.04(-0.09~0.18)	0.23(-0.16~0.61)	0.05(-0.23~0.34)
TSH	0.002(-0.05~0.06)	0.06(0.01~0.11) [a]	0.05(-0.08~0.18)	-0.14(-0.53~0.26)	-0.13(-0.57~0.31)
FT_4	0.001(-0.05~0.06)	0.001(-0.05~0.06)	-0.01(-0.14~0.12)	0.22(-0.33~0.77)	-0.13(-0.52~0.25)

TSH, Thyroid-stimulating hormone; FT4, Free thyroxine; FT3, Free triiodothyronine. CI, confidence interval.

Model is adjusted for age, gender, education.

[a] Indicates the correlation is significantly (P < 0.05).

Table S6 The proportion of cognitive impairment in different group

Group	N	NC	CI	χ^2/P
All Participants	1235	855(69.2)	380(30.8)	
Euthyroidism	957	660(69.0)	297(31.0)	0.007/0.93

Data are presented as percentages (%) for categorical variables. NC, Normal cognition; CI, Cognitive impairment.
